# Systematic Biases in Human Heading Estimation

**DOI:** 10.1371/journal.pone.0056862

**Published:** 2013-02-15

**Authors:** Luigi F. Cuturi, Paul R. MacNeilage

**Affiliations:** 1 German Center for Vertigo and Balance Disorders, University Hospital of Munich, Munich, Germany; 2 Graduate School of Systemic Neurosciences, Ludwig-Maximilians University, Munich, Germany; University of Muenster, Germany

## Abstract

Heading estimation is vital to everyday navigation and locomotion. Despite extensive behavioral and physiological research on both visual and vestibular heading estimation over more than two decades, the accuracy of heading estimation has not yet been systematically evaluated. Therefore human visual and vestibular heading estimation was assessed in the horizontal plane using a motion platform and stereo visual display. Heading angle was overestimated during forward movements and underestimated during backward movements in response to both visual and vestibular stimuli, indicating an overall multimodal bias toward lateral directions. Lateral biases are consistent with the overrepresentation of lateral preferred directions observed in neural populations that carry visual and vestibular heading information, including MSTd and otolith afferent populations. Due to this overrepresentation, population vector decoding yields patterns of bias remarkably similar to those observed behaviorally. Lateral biases are inconsistent with standard Bayesian accounts which predict that estimates should be biased toward the most common straight forward heading direction. Nevertheless, lateral biases may be functionally relevant. They effectively constitute a perceptual scale expansion around straight ahead which could allow for more precise estimation and provide a high gain feedback signal to facilitate maintenance of straight-forward heading during everyday navigation and locomotion.

## Introduction

As we move through the world we must constantly evaluate our heading direction in order to control where we are going. Heading can be estimated from both visual and non-visual sensory information. The most well studied visual cue to heading is the focus of expansion (FOE) of the optic flow field [1]. The predominant non-visual cue to heading is inertial force transduced by the otoliths of the vestibular system which indicate in which direction the body has been accelerated. Much research has been devoted to characterizing behavioral and physiology responses to such heading stimuli. Both humans and monkeys are more sensitive to visual than non-visual heading cues with minimum discrimination thresholds on the order of ∼1° and ∼4°, respectively [2]–[4]. Neurons responsive to both visual and non-visual heading stimuli have been identified in multiple brain regions [5]–[8].

Despite extensive research on heading sensitivity, the accuracy of both visual and non-visual heading estimates has not yet been systematically characterized. Two prior studies report some tendency for visual heading estimates to be underestimated in a limited range around straight ahead [9], [10] during pure linear motion. To our knowledge, published data on the accuracy of non-visual heading estimates is similarly very limited [11], [12]. Here we address these gaps in knowledge.

Accuracy and precision of both visual and non-visual heading estimates are measured for a 360° range of motion in the earth-horizontal plane. Accuracy in particular is important to measure because systematic biases can illuminate mechanisms of sensory transduction, encoding, and decoding. For example, if biases result from sensory transduction mechanisms, we might expect different patterns of bias to be observed for visual and vestibular heading estimates. Alternatively, systematic biases can also reveal assumptions that impact decoding of sensory stimuli. For example, the most common heading direction is straight forward and the nervous system might exploit this prior probabilistic information in a Bayesian fashion. In this case, we would expect both visual and vestibular heading estimates to be similarly biased toward the most common straight forward heading angles.

Results presented here show that both visual and vestibular heading estimates are biased and the patterns of bias are similar, perhaps suggestive of a common origin or cause. However, estimates are biased away from rather than toward the most common straight forward heading direction. Population vector models using physiological data from area MSTd and the otolith afferents can reproduce certain features of the bias data, providing a possible neurophysiological explanation. Preliminary aspects of this work were presented in abstract form [13].

## Materials and Methods

### Subjects

Fourteen healthy subjects (seven males) 20–31 years old participated in the study. All but one were naïve to the aims of the study. Subjects had no history of neurological, visual, or vestibular sensory disorders and had normal or corrected-to-normal vision.

### Ethics statement

Informed written consent was obtained from all subjects and all procedures were approved by the ethics committee of the University Hospital of Munich.

### Equipment

Vestibular experiments were conducted using a 6-degree-of-freedom motion platform (Moog© 6DOF2000E). Subjects were seated in a padded racing seat mounted on the platform. Their head was positioned against a vacuum pillow shaped according to subjects' head and their forehead was held with a padded strap to the chair. Sounds from the platform were masked by playing white noise in noise canceling headphones worn by subjects while performing the task. All experiments were conducted in a darkened room. During vestibular experiments, subjects either wore a blindfold (control procedure) or closed their eyes (identification task) during stimulus presentation.

During the visual experiment, subjects were seated on the motion platform but it did not move. The optic flow stimulus was presented on a stereo display (JVC©- GD-463D10, Refresh rate: 60 Hz) with dimensions 101.8×57.3 cm located ∼42 cm in front of the eyes, yielding a ∼107×∼75 degrees of visual angle field of view. The scene was rendered stereoscopically and viewed through polarized glasses (i.e. passive stereo). The field of view through the glasses was ∼107°×∼92°. Due to near viewing distance, a blurring film was placed over each lens of the glasses to blend neighboring pixels and weaken accommodative cues to screen distance. The visual scene was rendered using OpenGL© and consisted of a 3-dimensional volume (130×100×130 cm) of randomly placed, world-fixed, frontoparallel triangles with a base and height of 0.5 cm at a density of 0.01 triangles/cm^3^.

**Figure 1:**
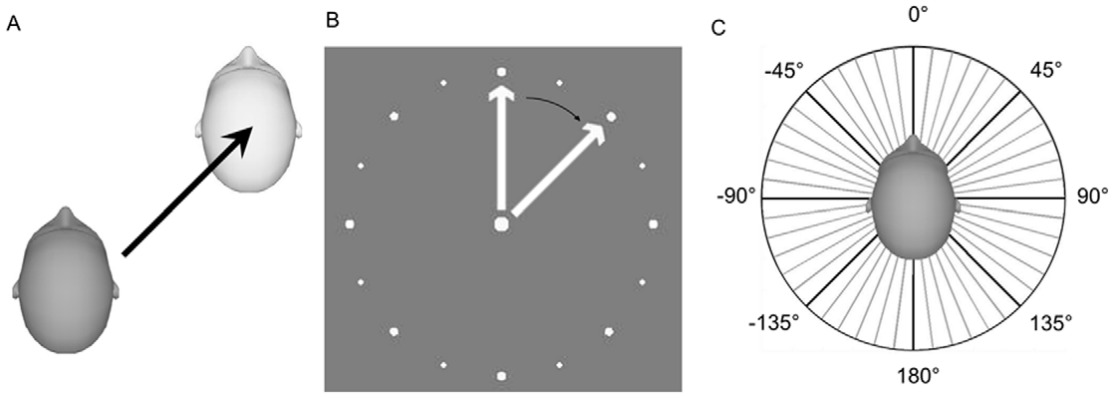
Illustrations of experimental procedures. **A)** Example movement. On each trial, subjects experienced a movement in the horizontal plane, for example 45°, as shown here. **B)** Response dial for identification task. Subjects indicated their heading direction after each movement by adjusting the orientation of an arrow within a dial on the screen. The setting shown here matches the movement from panel A). **C)** Investigated heading directions for the identification procedure (grey) and the control procedure (black).

Responses in all experiments were collected using a wireless numeric keypad. In two experiments (identification procedures) subjects used key presses to adjust the angle of a visual arrow presented within a dial (Fig. 1B) on a separate visual response display. The response display was positioned above the subjects' laps, oriented at ∼45° angle such that they could easily view both stimulus and response displays during the visual experiment. Viewing distance to the 22-inch display was ∼47 cm. The dial subtended ∼9° of visual angle. In two subjects, the large display was used in the vestibular condition such that the dial subtended ∼11°.

### Vestibular and Visual Heading Identification

On each trial, subjects experienced a passive visual-only or physical-only translation stimulus in a random heading direction in the earth-horizontal plane. The subjects pushed a button to start each trial. Each 1 s translation had a Gaussian speed profile, a displacement of 13 cm, peak velocity of ∼0.3 m/s, and peak acceleration/deceleration of ∼1.13 m/s^2^. After the movement they indicated their heading direction by adjusting the orientation of an arrow on the response display (see Figure 1A and 1B). They could adjust orientation quickly with one set of buttons (±1° per registered key press) or slowly with another (±0.1° per registered key press) to allow careful, precise indications. Once the subjects finished the adjustment they pressed a button to register the response and then the next trial began after another button press. The procedure consisted of 5 blocks, each containing two repetitions of 48 different heading directions (0° to 352.5°, step size 7.5°) and lasting ∼20 minutes. Presentation order was randomized across blocks and across subjects.

Visual and vestibular procedures were run separately. Six subjects (24–31 years old, 3 males) participated in the both procedures; 4 of them performed the vestibular procedure first. In order to familiarize these subjects with the task of judging self-motion direction from the visual-only stimulus, they were first presented with a short (∼5 min) training session in which vestibular and visual stimulation were delivered simultaneously and they were asked to verbally report the perceived direction (either forward, backward, leftward, or rightward).

### Control Procedure - Vestibular Heading Discrimination

In the identification procedure described above subjects adjusted the orientation of a visual probe on the screen to indicate their perceived heading, Bias or inaccuracy in observed responses could reflect response biases inherent to the visual adjustment task, rather heading bias. Vestibular heading biases were therefore also measured using the discrimination procedure described below for comparison. Twelve subjects (20–31 years old, 6 males) participated in both the vestibular identification and discrimination procedures.

On each trial, subjects were passively translated in the earth-horizontal plane (same 1 s profile described above). The subjects pushed a button to start each trial and then performed a 2-alternative-forced-choice task in which they indicated with a button press if their heading direction was clockwise or counter-clockwise compared to the investigated heading direction. The investigated heading direction was fixed in a given block. To ensure subjects properly understood which direction was being investigated they were shown a schematic similar to Fig. 1C before each block with only the investigated heading direction for that block depicted.

Heading direction of the stimulus was varied from trial to trial according to a staircase procedure in order to find the physical heading that was perceived to be equal to the subject's internal representation of the given investigated heading for that block. Each block began with a one-up/one-down (1U1D) procedure. Heading angle of the stimulus was increased when the subject indicated that the previous heading angle was less than the investigated direction, and decreased when they indicated that it was greater. This 1U1D stepping rule converges to the 50% point of the psychometric function, the so-called point of subjective equality (PSE) where the physical heading is perceived to be equal to the investigated heading direction. There were 35 trials per staircase and step size was 4°. Two such staircases were interleaved in a given block; the descending and ascending staircases began at the investigated heading direction plus and minus 26°, respectively. The average of the staircase reversals (ignoring the first four) provides an estimate of the PSE.

Immediately afterwards, subjects completed a second staircase procedure for the same investigated heading direction with interleaved two-up/one-down (2U1D) and one-up/two-down (1U2D) staircases which converge to the 30% and 70% points of the psychometric function, respectively. The initial heading directions for the descending (1U2D) and ascending (2U1D) staircases were equal to the average PSE measured in the previous block plus and minus 18°, respectively. There were 40 trials per staircase and step size was 4°.

This two-session procedure lasted ∼45 minutes and was repeated for each of the 8 investigated heading directions, i.e. all of the cardinal (−90°, 0°, 90°, 180°), and inter-cardinal (−135°, −45°, 45°, 135°), directions (see Figure 1C). For each investigated heading direction a psychometric function was fit to all staircase data from both sessions to estimate the PSE and just-noticeable difference (JND). The order in which the different investigated heading directions were tested was randomized across subjects.

### Data Analysis

For the identification procedures, perceived heading for each subject and heading direction (0° to 352.5°, step size 7.5°) was calculated as the circular mean of the indicated direction across the ten presentations (5 blocks X 2 presentations per block). Outliers clearly resulted from subjects accidentally registering their response before they had finished (or even begun) adjusting the angle of the arrow. Consequently we adopted the Chauvenet method for outlier exclusion: Responses were excluded if they deviated more than 2.4 standard deviations from the mean of the ten presentations. In total we excluded 84 out of 5760 measurements (1.45%) in the vestibular experiment with twelve subjects, and 103 out of 2880 measurements (3.57%) in the visual experiment. Additional outliers in the visual experiment resulted from subjects occasionally indicating the direction of the visual motion pattern which is exactly opposite the self-motion direction.

For the control procedure, data were analyzed separately for each investigated heading direction. For each presented heading angle, we calculated the proportion of trials in which subjects reported the perceived angle to be clockwise from the investigated heading direction, and we fit a cumulative Gaussian to the data using the psignifit software package [14], [15]. PSE and JND are the mean and standard deviation of the cumulative Gaussian fit, respectively. Deviation of the PSE from the investigated heading direction provides a measure of the bias or accuracy of each subject's heading estimate. The JND provides a measure of the precision of each subject's heading estimate.

Results from the vestibular identification and control procedures were compared on a subject-by-subject, heading-by-heading basis to examine to what extent observed biases may be task-related. The PSEs estimated from the discrimination data provide a measure of the physical heading stimuli that give rise to perception of cardinal and inter-cardinal heading directions. However, these exact physical stimuli were not presented in the identification procedure. Perceived heading corresponding to these intermediate physical heading stimuli were therefore interpolated by fitting a 12-degree polynomial using the Matlab© polyfit function which performs a least squares fit. In this way it was possible to estimate from the identification data the physical stimuli that give rise to perception of cardinal and inter-cardinal heading directions.

Resulting bias values from vestibular identification and discrimination procedures were compared using Spearman correlation analysis (excluding 0° and 180°, which were unbiased). Visual and vestibular identification data was also compared using Spearman correlation. For all experiments, one-way ANOVA was used to examine the effect of heading direction on bias. A two-way repeated measured ANOVA with heading and condition as factors was also used to compare data from vestibular and visual heading identification tasks. T-test was used to test whether each bias value was significantly different from zero. Paired student T-test was used to compare data from vestibular and visual identification tasks and to examine whether biases were symmetric for leftward and rightward movements. When appropriate, the Bonferroni correction for multiple comparisons was applied. Wilcoxon signed-rank test was used to examine if biases were consistently positive or negative across subjects in each experiment.

### Population vector decoding

Given biases observed for heading perception, we considered plausible neurophysiological mechanisms that could give rise to such biases. In particular, we consider decoding algorithms that could be applied to derive biased perceptual estimates. The well-known population vector decoder [16] generates biased output when distributions of preferred directions in the population are not uniform, with results being biased toward directions that are overrepresented by the population [17]. Distributions of preferred visual and vestibular heading directions of MSTd neurons are non-uniform and Gu et al [18] point out that a population vector decoding of 800+ MSTd neurons leads to biased heading estimates similar to the biases observed here. Agreement between our results and population vector prediction is evaluated with Spearman correlation and R^2^ statistics.

However, biased perception may arise earlier in the stream of vestibular processing. We therefore sought to apply a similar analysis to vestibular otolith afferent population data published by Fernandez and Goldberg [19]. They represent preferred direction as the polarization vector of each neuron. Similar to MSTd neurons, these preferred directions are also distributed non-uniformly in the head-horizontal plane. For this population of 313 neurons we calculate the mean firing rate (*d*) in response to a heading stimulus as *d* = *s* (*F·P*)+*d_0_* [19]. *F* is the total force which is the sum of gravity and linear acceleration heading vectors (*G*+*I_h_*). *P* is the unit polarization vector, *s* is the sensitivity, and *d_0_* is the resting discharge[19]. Because each neuron provides information about both the acceleration and deceleration phases of a transient movement, we quantify the information conveyed by each neuron as the peak-to-trough modulation in firing rate. In other words, we calculate the response as the firing rate at the point of maximum acceleration minus the firing rate at the point of maximum deceleration. This scalar response is multiplied by the polarization vector of each neuron and these vectors are summed to generate the population vector estimate for each heading stimulus. The decoded heading estimate is the angle of the projection of this vector onto the horizontal plane.

### Bayesian prior probability model

Biases can be explained in the Bayesian framework as the consequence of an optimal estimation strategy whereby the brain combines probabilistic sensory and prior information in a statistically optimal fashion [20]. We therefore sought to determine the shape of the prior distribution that is most consistent with our observed data. We began with the observation that the pattern of biases from the identification experiments appeared roughly consistent with a prior distribution composed of two Gaussians with peaks for lateral movement directions (i.e. +/−90). Then our goal was to find the spread (σ_prior_) of these Gaussians that can account best for our data (assuming they have the same spread).

In our simple Bayesian model, for a given heading stimulus, we multiply the likelihood distribution for that stimulus times the prior distribution to generate the posterior distribution. The perceptual estimate is based on the maximum of the posterior distribution. We assume the likelihood distribution is unbiased (i.e. the mean of the distribution is equal to the angle of the presented heading stimulus) and Gaussian in shape with standard deviation that increases with increasing heading eccentricity, as previously reported [18].

Thus, the only free parameter was the spread of the prior distribution, σ_prior_. For each candidate σ_prior_, we calculated estimated heading directions (and thus biases) predicted by this prior for each heading stimulus. These predictions were compared to our data until we found the best value of σ_prior_ in a least-squares sense.

## Results

Visual and vestibular heading biases were measured in the earth-horizontal plane. Subjects experienced a movement and then indicated the direction of the movement by adjusting the angle of an arrow within a dial on the response display. Results from visual and vestibular conditions are compared to evaluate to what extent inaccuracies might result from modality-independent mechanisms. Results are also compared to those from a control task to evaluate task-dependent response biases. Finally, biases are compared to those predicted based on a population vector decoding of MSTd and vestibular afferent neural populations.

### Vestibular heading biases

**Figure 2:**
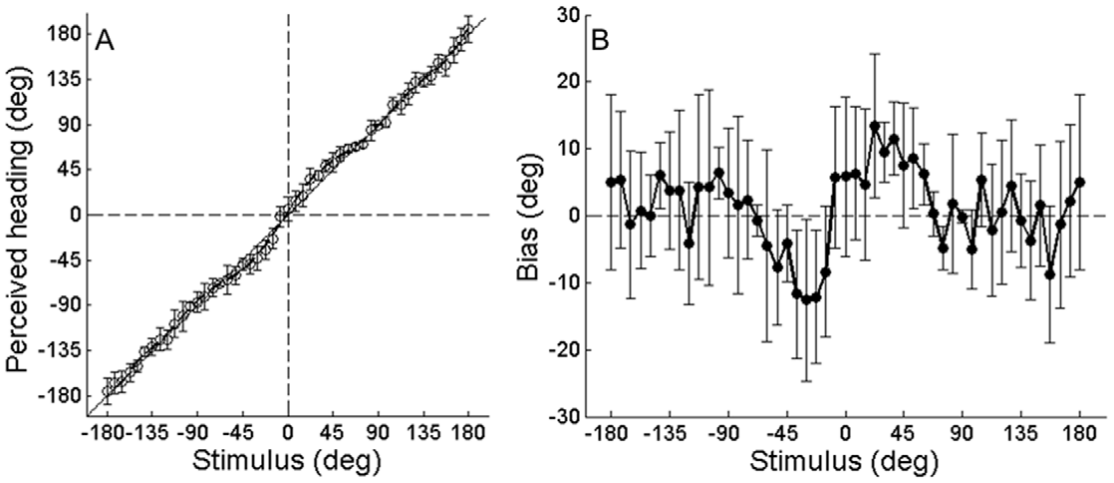
Vestibular identification results for an individual subject. **A)** Mean (+/− SD) angle indicated in the identification task in response to each heading stimulus. A polynomial function was fit to the data to allow interpolation of expected response for intermediate stimulus values. **B)** Data from A) replotted to illustrate bias (perceived heading minus stimulus) for each presented heading angle.

Vestibular estimates were often inaccurate or biased. This is illustrated by the points in Fig. 2A which plot the mean (+/− SD) indicated heading direction (y-axis) for each presented heading stimulus (x-axis) for one individual subject. These points clearly deviate from the solid black line with unity slope. Corresponding bias values, calculated as the mean perceived heading direction minus the actual direction of the presented heading stimulus, are shown in Fig. 2B. This subject systematically overestimated the actual heading angle by 5° to 15° for heading stimuli between +/−15° and 60°.

**Figure 3:**
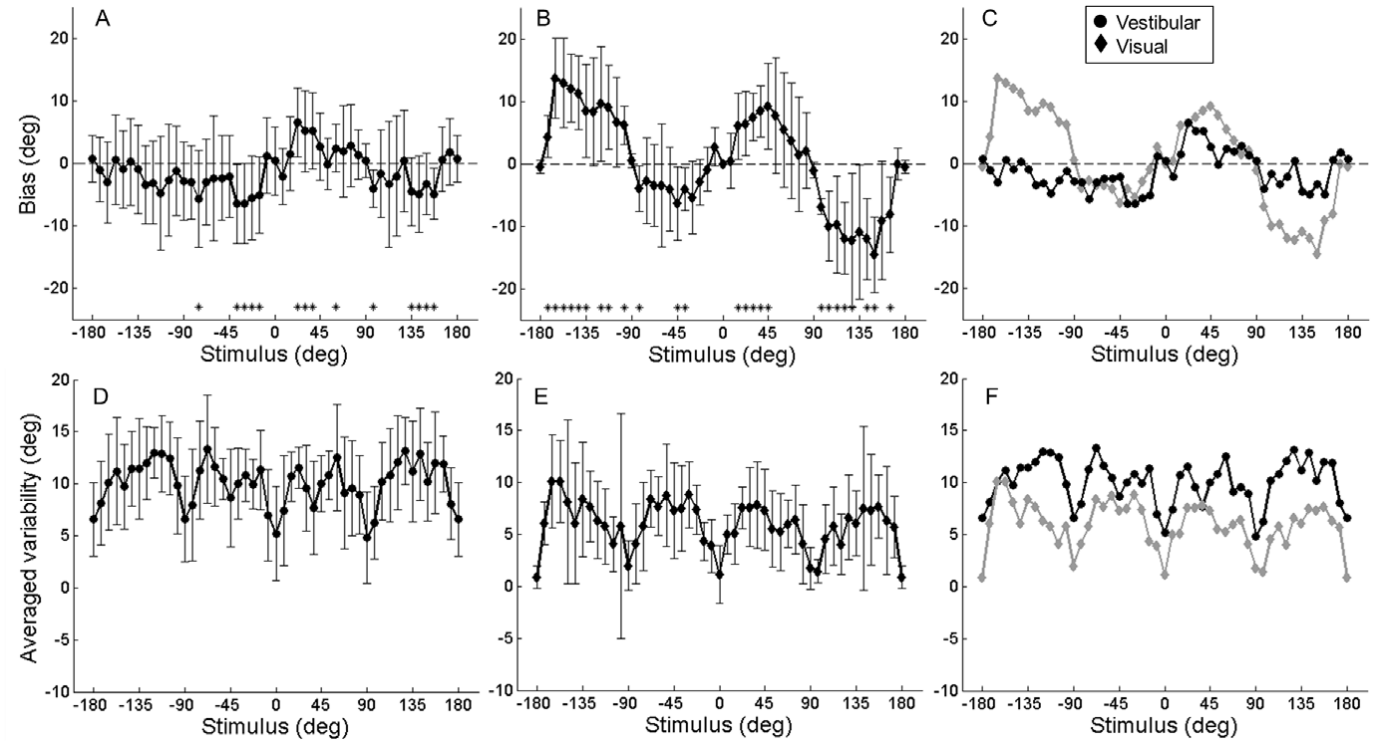
Visual and vestibular bias (top row) and variability (bottom row) across modalities. Error bars represent SD. **A,** **D)** Vestibular identification procedure. **B,** **E)** Visual identification procedure. **C)** Data from A) and B) re-plotted without error bars to facilitate comparison. **F)** Data from D) and E) re-plotted without error bars to facilitate comparison. Note, asterisks indicate heading angles for which bias was *most* significant, i,e, p<0.05 before Bonferroni correction. The correction is not applied here for illustrative purposes only.

A similar pattern of results is observed across subjects (Fig. 3A). Bias depends significantly on heading direction (*F* (47,11) = 3.05, *p*<0.001). Those heading directions with the most significant biases are indicated by an asterisk in Fig. 3A. Overestimation was observed symmetrically for both leftward and rightward forward movements between +/−22.5° and 37.5°. The largest biases are on the order of ∼|6|° and are also observed in this range. Underestimation is observed only for backward and rightward movement directions (Fig. 3A, heading angle >90°). However, paired t-tests corrected for multiple comparisons did not indicate a significant left-right asymmetry.

While our main focus is to examine accuracy of heading estimates, we also obtained information about precision (or variability) of heading estimates via the standard deviation of the indicated heading direction (10 repetitions per subject) for each presented angle. The average variability of the indicated heading direction across subjects is shown in Fig. 3D. Mean variability depended significantly on heading direction (*F* (47,11) = 3.69, *p*<0.001). It is interesting to note that variability is minimal for the cardinal directions and higher for in between directions. Similar results are often observed in other domains and these are referred to as oblique effects [21]. Variability also appears markedly reduced close to +/−45°, but not +/−135°.

### Visual heading biases

Visual heading biases were investigated using the same experimental and analysis methods described above except that subjects judged heading based on visual optic flow patterns rather than real physical movements. Across subject results reveal substantial over- and underestimation of heading angle for forward and backward movement directions, respectively (Fig. 3B). Bias depended significantly on heading direction (*F* (47, 5) = 8.19, *p*<0.001).

The average variability of the indicated heading direction across subjects (Fig. 3E) was similar to that observed in the vestibular identification task. Mean variability depended significantly on heading direction (*F* (47,5) = 1.83, *p*<0.01). There was no evidence to reject the hypothesis that variability is symmetric for leftward and rightward movements. These measures of increasing visual heading variability as a function of heading eccentricity during forward movement are similar to previous reports [18], [22].

### Vestibular and visual biases compared

**Figure 4:**
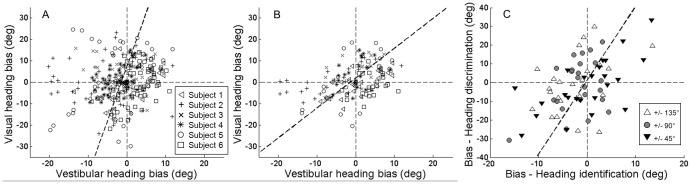
Biases compared across tasks and modalities. The dotted lines represent two-way least-squares fits. **A)** Visual and vestibular identification biases compared for all heading directions for six subjects. **B)** Same but for forward movements only. **C)** Vestibular identification and discrimination biases compared for heading angles close to +/−45°, +/−90°, and +/−135°, for twelve subjects.

Vestibular and visual biases are compared to examine the extent to which they may be explained by common, multimodal biases in underlying spatial processes versus modality specific sensory processes (Fig. 3C). Results are similar for forward movement directions, but differ markedly for backward movement directions. Repeated measures ANOVA across the six subjects who participated in both tasks did not reveal an overall significant effect of modality (*F* (1,5) = 1.36, *p* = 0.29). Nevertheless, a significant interaction (*F* (47,5) = 5.5, *p*<0.001) suggests that the effect of heading *F* (47,5) = 5.56, *p*<0.001) depended at least partially on modality. When the analysis is applied to forward directions only, the interaction becomes non-significant (*F* (22,5) = 1.5, *p* = 0.08), whereas analysis of backward only heading directions shows a significant interaction (*F* (22,5) = 8.12, *p*<0.001),

Visual and vestibular biases are further compared by plotting these values versus one another for all subjects and heading angles (Fig. 4A). The slope of the line fit to the data (type II regression) is 3.72, consistent with the observation that visual biases are generally greater than vestibular biases, particularly for backward movement directions. This difference is significant (*p*<0.01, paired t-test). Nevertheless, a weak but significant positive correlation is observed (ρ = 0.23, *p*<0.001). When only forward movements are examined, a significantly greater correlation is observed (ρ = 0.45, *p*<0.001; Fisher's r-to-z transformation: z-score = 2.369, 2 tail *p*<0.05) and the slope of the line fit to the data decreases to 1.39 (see Figure 4B), indicating a closer relationship. When considering only backward movements, the correlation is not significant (ρ = 0.04, *p* = 0.59). A parsimonious explanation is that biases across modalities result primarily from inaccuracies in underlying spatial processes, with additional modality-specific effects manifest particularly for backward heading directions.

Variability of visual and vestibular identification data are also compared (Fig. 3F). Visual variability is consistently smaller than vestibular (*F* (1,5) = 44.09, *p*<0.01), consistent with prior reports that visual heading estimates with 100% percent motion coherence are generally more reliable than vestibular [2]. Despite differences in magnitude, visual and vestibular variability show remarkably similar dependencies on heading angle, to the extent that the oblique effects observed in the vestibular heading identification task are replicated in the visual task. This similarity in the effect of heading angle is confirmed by the absence of an interaction between modality and heading factors (*F* (47,5) = 0.86, *p* = 0.72).

### Control Procedure - Vestibular Heading Discrimination

To examine the possibility that observed biases depend on the arrow-setting response task used in the identification procedure rather than the heading estimation process itself, vestibular heading estimation was additionally assessed using a discrimination task. In this control procedure, subjects experienced a movement in darkness and then indicated via button press if the movement was clockwise or counter-clockwise relative to a given reference direction. Results identify the physical heading that is perceived to be equal to each of the eight reference directions (Fig. 1C, black). Observed biases are compared across the twelve subjects who performed both vestibular identification and discrimination tasks.

**Figure 5:**
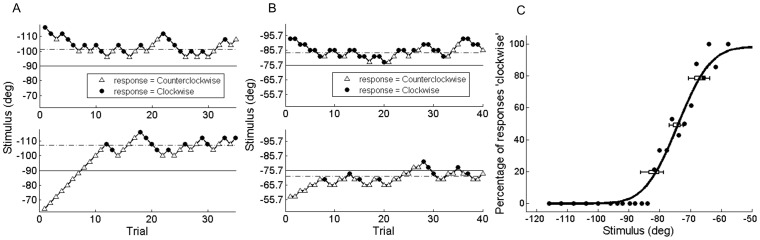
Vestibular discrimination results for one subject at one investigated direction (−90°). **A)** Trial history for 1U1D staircase block. Upper and lower panels represent the two interleaved staircases that converged from above and below the investigated direction. Dashed line indicates mean of staircase reversals which approximates the PSE. **B)** Trial history for 1U2D and 2U1D staircase block. Upper and lower panels represent the two interleaved staircases that converged from above and below the mean PSE from the 1U1D block. **C)** Psychometric function fit to all data from the staircase blocks shown in A) and B). Error bars show 95% and 99% confidence intervals of the fit at the 20%, 50%, and 80% correct points.

Example data for one individual subject and one investigated heading direction (−90°) are shown in Fig. 5. Each investigated direction was tested in two separate blocks, one composed of two interleaved 1U1D staircases (Fig. 5A), and a second composed of two interleaved 2U1D, 1U2D staircases (Fig. 5B). A cumulative Gaussian function (Fig. 5C) was fit to this data to obtain an estimate of the mean (PSE) and standard deviation (JND) for each investigated heading direction. The PSE indicates the physical heading direction that is perceived to be equal to the investigated heading direction. For example, the data in Fig. 5C yield a PSE of −75°, meaning that when a stimulus of −75° was presented this subject perceived a heading direction of −90°, i.e. overestimation of 15°. The JND provides an estimate of the precision or variability of the heading estimate, 8.42° in Fig. 5C. This procedure was repeated for all the investigated heading directions (Fig. 1C, black).

**Figure 6:**
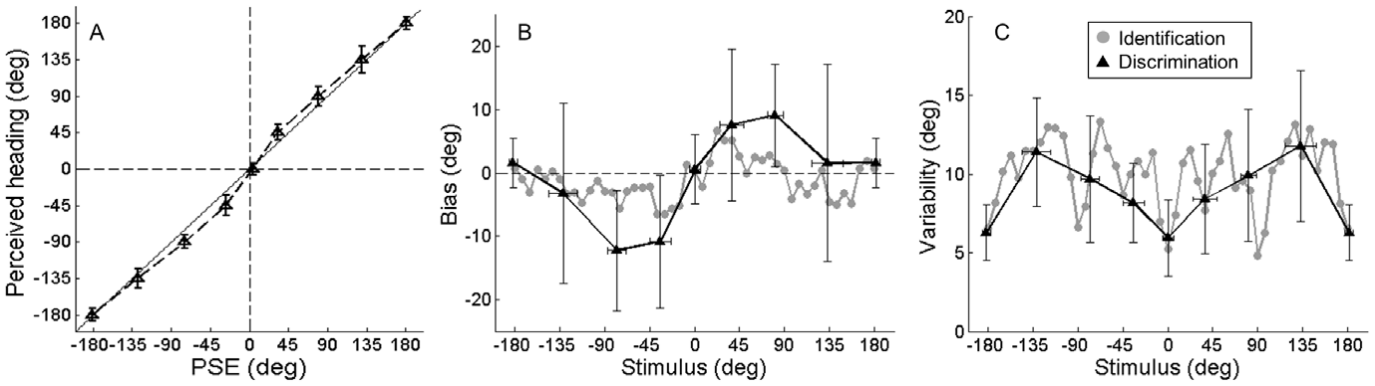
Vestibular discrimination results. **A)** Example results for an individual subject shown in the same format used for the individual identification data (Fig. 2A). Plot illustrates the physical stimulus (x-axis, PSE) perceived equivalent to each investigated reference direction (y-axis). Error bars represent JNDs. **B)** Mean (+/−SD) bias across subjects in the discrimination procedure (black). Horizontal error bars represent SD of the PSEs. For comparison, mean bias in the identification procedure (grey) is replotted from Fig. 3A. **C)** Mean (+/−SD) variability (i.e. JND) across subjects in the discrimination procedure (black). Horizontal error bars represent SD of the PSEs. For comparison, mean variability in the identification procedure (grey) is replotted from Fig. 3D.

Individual subject results (Fig. 6A) reveal overestimation of heading angle during forward movement similar to that observed for the identification task (Fig. 2A). Across all subjects (Fig. 6B, black), the largest average biases reflect overestimation on the order of ∼|10|° and are observed for the investigated directions +/−45° and +/−90°. Bias depended significantly on heading direction (*F* (7,11) = 3.24, *p*<0.01)), and biases for investigated heading directions −90°, −45°, and 90° were significantly different from zero (p<0.01). There is no evidence to reject the hypothesis that bias is symmetric for leftward and rightward movements (paired t-test, p>0.05 for all angles).

In general, similar patterns of bias were observed in the identification and discrimination tasks (Fig. 6B, black versus grey), with the exception that much larger biases were observed in the discrimination task for the investigated directions of +/−90°. Direct quantitative comparisons between the two tasks can only be made for directions that were investigated in both tasks, and this is limited by the discrimination task, for which the sampling of investigated direction was sparser. Individual results of the discrimination task provide a measure of bias (investigated direction – PSE) in perceived heading direction for a particular physical heading stimulus (the PSE). For example, as described previously, the results shown in Fig. 6A indicate a bias of −15° for a physical heading stimulus of −75°. Thus, the relevant value for comparison from the identification task is also the bias observed for that particular physical stimulus (the PSE, e.g. −75°), which can be read off from the function fit to the individual subject identification task data (see Fig. 2A). In this way, 6 pairs of bias measurements were obtained for each subject corresponding roughly to perceived directions of +/−45°, +/−90°, and +/−135°. Little bias was observed for headings 0° and 180°, so they were excluded from the comparison analysis.

Resulting pairs of measurements are plotted versus one another in Fig. 4C. The slope of the line fit to the data (type II regression) is 3.94, indicating that discrimination task biases are generally greater than identification task biases. This difference is significant for heading +/−90° (paired t-test). Nevertheless, a significant positive correlation is observed (ρ = 0.43, *p*<0.01) suggesting that heading biases follow a similar trend for the two tasks.

Precision of vestibular heading estimates also shows a similar pattern across procedures (Fig. 6C). Average JNDs across subjects are plotted in Fig. 6C versus the average PSEs (i.e. the actually presented heading stimulus) for each investigated heading direction. JND depends significantly on investigated heading direction (*F* (7,11) = 5.72, *p*<0.001). As may be expected, the lowest mean variabilities are observed for heading directions 0° and 180° while the highest mean variabilities are observed for the ecologically uncommon heading directions of +/−135°. The JNDs measured here are similar in magnitude to those measured previously using a 2-alternative-forced-choice task [18]. Data from that study also exhibit an approximately linear increase in JND with increasing heading eccentricity during forward self-motion. Due to the sparse sampling, it is not possible to confirm if an oblique effect pertains to results from this task, as to results of the Identification task. The close correspondence between variability measured in these two tasks (Fig. 6C; paired t-test, *p* = 0.32), as well as agreement with prior reports [18] suggest that variability in both tasks arises primarily from noise on the vestibular heading estimate, with little noise added by the response process, whether it be setting a visual arrow or comparing to an internal standard.

### Population vector decoding

A population vector decoding of a neural population with non-uniform preferred directions can lead to biased estimates [17]. Here we calculate biases predicted by a population vector decoding of vestibular otolith afferent data from Fernandez & Goldberg [19] and compare these predictions with our data.

**Figure 7:**
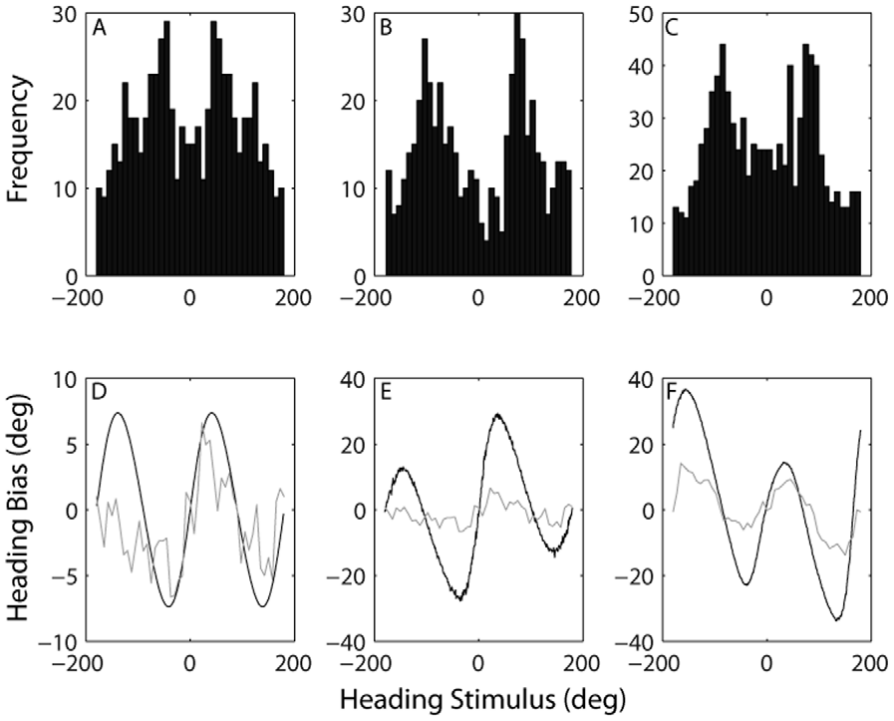
Distribution of preferred directions (top row) and resulting population vector decoding predictions (bottom row). **A)** Preferred directions of otolith afferents. **B)** Preferred directions of MSTd neurons for vestibular heading stimuli. **C)** Preferred directions of MSTd neurons for visual heading stimuli. **D)** Afferent predicted (black) and observed (grey) vestibular bias. **E)** MSTd predicted and observed vestibular bias. **F)** MSTd predicted and observed visual bias. Note, panels B), C), and predictions in E), and F) reproduced from Gu et al 2010.

The distribution of preferred directions (i.e. polarization vectors) projected on the horizontal plane is illustrated by the histogram in Fig. 7A. This distribution is symmetric because Fernandez & Goldberg [19] report all polarization vectors in coordinates of the left labyrinth, and we mirror this population to simulate an overall bilateral population. The distribution is non-uniform and shows peaks close to +/−50 and troughs close to 0 and 180.

The predicted bias is illustrated by the black line in Fig. 7D. Due to the symmetrical left/right distribution of preferred directions, the predicted bias is least for forward and backward movement directions. And because there are more neurons with preferred directions closer to lateral (i.e. +/−90°) than forward movement, estimates are biased toward lateral directions and away from forward/backward ones. This is similar to the bias observed across 12 subjects in the vestibular identification experiment in our study, shown by the grey line. The population vector prediction replicates the overall pattern of results, as indicated by the positive, significant correlation between predicted and observed biases (ρ = 0.59, p<0.001). However, due to asymmetry of observed biases for backward movements, the match is much better for rightward (ρ = 0.78, p<0.001) than leftward (ρ = 0.39, p = 0.06) directions. Overall goodness-of-fit (R^2^ = −1.07), which depends on bias magnitude, is also better for rightward (R^2^ = −0.10) than leftward (R^2^ = −5.05) movement directions.

For comparison we also show predictions of visual and vestibular heading bias based on population vector decoding of over 800 MSTd neurons reproduced from a previous report (Gu et al [18]. Distribution of preferred directions is non-uniform for both vestibular (Fig. 7B) and visual (Fig. 7C) sensitivity, with peaks close to +/−90° and troughs close to 0 and 180. Resulting biases generated by these non-uniformities are shown in Fig. 7E and 7F, respectively. Again, predicted and observed biases are very well correlated in both visual (ρ = 0.89, p<0.001) and vestibular (ρ = 0.74, p<0.001) experiments, but the magnitude of the bias is not as well predicted (R^2^ = −2.86 for visual, R^2^ = −18.04 for vestibular). It is worth emphasizing that the close correspondence between observed behavior and the neural population vector predictions is all the more remarkable because all predictions were generated with no free parameters.

### Best-fitting prior distributions

Biases have often been described to result from statistically optimal combinations of noisy sensory estimates with prior knowledge, represented by prior probability distributions [20], [23]. We therefore estimated the shapes of the prior probability distributions that would be most consistent with the patterns of bias observed in the vestibular and visual identification procedures.

**Figure 8:**
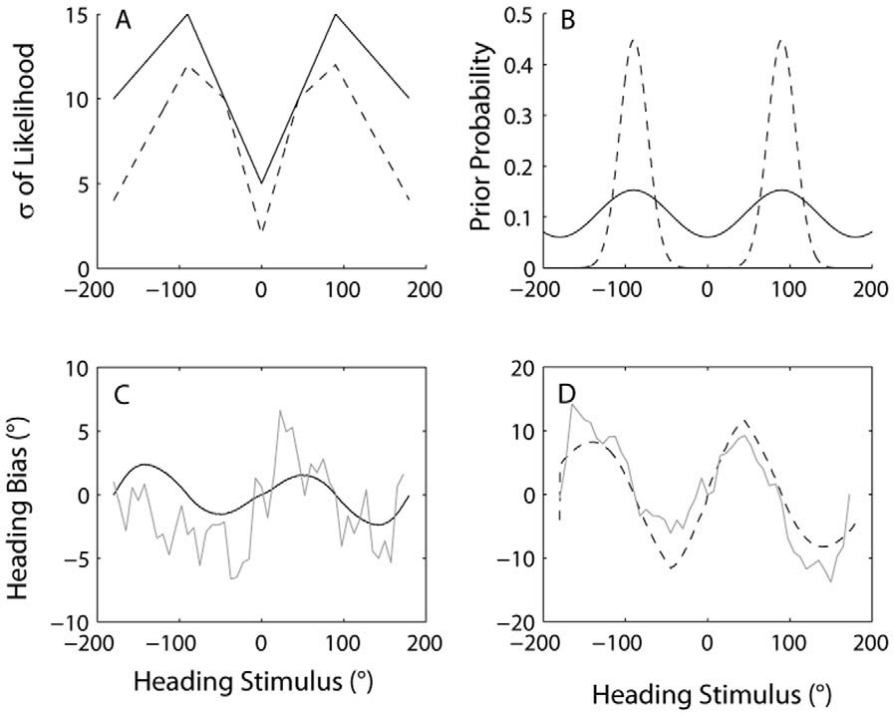
Bayesian model and predictions. **A)** Standard deviation of visual (dotted) and vestibular (solid) likelihoods as a function of heading angle used in the model (adapted from Gu et al. 2010). **B)** Best-fitting prior distributions for visual and vestibular identification data. Each curve is a sum of two Gaussians centered at +90° and −90°, with equal SD. σ_prior_ equals 17° and 50° for visual and vestibular priors, respectively. **C)** Predicted (black) and observed (grey) vestibular biases. **D)** Predicted and observed visual biases.

Our simple Bayesian model assumes that the noise on the vestibular and visual sensory estimates depends on heading angle. The exact values used for the standard deviation of the likelihood are shown in Fig. 8A; these values are duplicated roughly from Gu et al [18], Fig. 2A. Note, to our knowledge, aside from the current study, there is no published data on variability of visual heading judgments for backward heading directions, i.e. angles between +/−90 and 180°. Therefore we extrapolated what seem to be reasonable threshold values for these directions.

Given our assumptions (see Methods) the best-fitting priors for vestibular and visual heading estimation are shown by the solid and dashed lines in Fig. 8B, respectively. The standard deviations of the Gaussians (σ_prior_) are 50° and 17°, respectively. The biases predicted by these priors are illustrated in Figs. 8C and D, respectively.

Note that a much better fit is obtained for the visual (R^2^ = 0.76) than for the vestibular (R^2^ = 0.06) data. This is because the Bayesian model is left/right symmetric, and it is therefore able to fit the symmetric biases observed for the visual experiment much better than the asymmetric biases observed in the vestibular one. If the model fit to vestibular data is applied only to the rightward movement directions, a better fit (R^2^ = 0.54) and a smaller value of σ_prior_ (43°) is obtained.

## Discussion

Heading angle is consistently overestimated during forward self-motion. This novel finding applies to both visual and vestibular heading estimation, and it is observed for both response methods tested here. Such biases are surprising given the behavioral importance of heading estimation for effective navigation, including safety during vehicle guidance. Parameter-free population vector decoding of vestibular afferent and MSTd neural populations predict biases that are remarkably similar to those observed experimentally and therefore constitute a plausible physiological mechanism underlying these biases. This pattern of bias is inconsistent with a Bayesian prior for the most common straight-forward heading direction, and may instead represent a perceptual scale expansion [24] to facilitate heading discrimination near straight ahead.

### Heading bias and possible neural mechanisms

Similarity of visual and vestibular biases may be due to common underlying biases in multimodal heading representations. Neurophysiological studies using microstimulation [25],calculation of choice probability [8], and population modeling [26] suggest that both visual and vestibular heading perception is mediated by area MSTd. Therefore, we compare our results to predictions based on population vector decoding of MSTd activity.

Gu et al [18] showed that a population vector decoding of more than 800 MSTd neurons yields visual and vestibular heading biases similar to those reported here. The distributions of preferred directions is non-uniform with more neurons preferring lateral than fore-aft movement directions (Fig. 7B,C). This leads to biased estimates (Fig. 7E,F). The pattern of results is generally very consistent with the behavioral data, with strong correlations observed between predicted and observed biases (visual ρ = 0.89, p<0.001; vestibular ρ = 0.74, p<0.001). In particular, there is an uncanny resemblance between predicted and observed visual biases, both of which are greater for backward than forward heading directions. Nevertheless, the magnitude of predicted biases is considerably greater than what we observed, particularly for the vestibular condition.

To investigate the possibility that biases might arise earlier in the stream of vestibular sensory processing, the population vector decoder was also applied to otolith afferent data. When otolith afferent polarization vectors are projected onto the horizontal plane, the distribution of preferred directions is also non-uniform (Fig. 7A), and therefore gives rise to biased population vector predictions (Fig. 7D). While the correlation is somewhat weaker (ρ = 0.59, p<0.001), the magnitude of biases predicted from the afferent population matches our behavioral data better than the prediction from MSTd population activity (R^2^ = −1.07 versus R^2^ = −18.04).

It is remarkable that such parameter-free decoding of neural activity both at the sensory periphery and more centrally can generate biases in the same direction and of similar magnitude to those observed empirically. These findings echo similar reports in other domains. For example, Girshick et al. [23] recently showed that biases in visual orientation judgments are well explained by a population vector decoding of V1 neural populations with non-uniform distributions of preferred directions.

### Theoretical explanations of heading biases

In addition to considering *how* such biases may be represented neurally, we also address the question of *why* heading perception is biased. Biased perception is sometimes modeled to result from a Bayes-optimal estimation strategy that combines sensory and prior information [20], [23]. For example, perception of body orientation is underestimated during large tilts (the Aubert Effect) and this can be explained by a Bayesian prior for the most common upright body orientation [27]–[30].

We therefore examined the prior distributions that can best explain our data. Forward movement is most common during our daily lives, but observed biases are consistent instead with priors peaked for lateral movement directions (Fig. 8). The priors serve to bias estimates away from straight ahead. Consequently, spatial representations are expanded for the most ecologically important forward heading directions. This expansion allows increased discriminability at the cost of overestimation for heading angles in the region close to straight ahead. Such perceptual scale expansion has recently been proposed as an efficient coding strategy for locomotor space [24].

In this context, priors can be conceptualized to represent subjects' tendency to categorize movements as either leftwards or rightwards. Indeed, categorical processing can account for spatial biases [31], and the use of Bayesian priors to implement categorical processing has been proposed previously [32]. Such left-right categorization can be considered a natural requirement for maintaining straight-forward heading. Functionally, this categorical processing and associated scale expansion could act to facilitate maintenance of straight-forward heading during navigation and locomotion by providing a high-gain feedback signal to rapidly correct unwanted deviations.

It is interesting to note that such Bayesian computations are not inconsistent with the neurophysiological observations described in the previous section. Recent work shows how Bayesian computations with prior probabilities, like those suggested here, can be implemented via a population vector decoding of neural populations with non-uniform preferred directions [23], [33].

### General biases in spatial processing

Alternatively, the common biases reported here could reflect inherent bias in human spatial processing more generally. For example, prior research suggests that spatial updating operates on an amodal spatial image [34], [35]. Heading judgments require mapping an egocentric stimulus into representation of movement relative to the world, and this process may rely on such a spatial image. In this case, one might predict similar biases in angular estimates across different modalities (e.g. audition) and tasks (e.g. target localization). Biases in auditory and visual localization have been measured and compared (e.g. [36]). However, the pattern of results varies depending on the response method employed, complicating comparisons across studies, tasks, and modalities. To best evaluate this hypothesis of general spatial biases it would be necessary to assess heading and localization using a common methodology.

### Task-specific response biases

It is well-known that behavioral responses can include bias resulting directly from the response measure itself. To evaluate response bias, we measured vestibular heading using both the identification task and a control procedure, the discrimination task. Biases across tasks were similar in terms of direction, but larger in the discrimination than in the identification task. In particular, in the discrimination task, heading angles near +/−80° were perceived equivalent to lateral movement (i.e. +/−90°), a bias of ∼10°, whereas a similar angle presented in the context of the identification task was biased by only ∼3° (see Fig. 6B). Such differences in magnitude must reflect task-specific factors.

For example, in the identification task it is likely that arrow-setting responses were drawn to the reference points around the circumference of the dial (Fig. 1B). This can explain the reduced bias and variability near the corresponding stimulus directions, e.g. +/−45° and 90° observed in both visual and vestibular identification results (Fig. 3). Also in the identification task, subjects probably assumed that stimulus directions were uniformly distributed in the earth-horizontal plane, and this would have led them to distribute their responses more uniformly, also reducing bias. In the discrimination task, on the other hand, we used a staircase procedure and individual heading angles were tested in separate blocks. Because of repeated movements in the same or similar direction, some adaptation may have occurred, and may have impacted heading bias. Unfortunately, it is difficult to conceive of response techniques that avoid response bias altogether. Nevertheless, the broad similarities in the pattern of results across the two tasks, particularly the overestimation of heading angles during forward movements, suggests a consistent underlying bias in the heading estimation process itself.

### Modality-specific sensory biases

While visual and vestibular biases are broadly similar, especially for forward movements, there are some notable differences. These differences result in rather weak correlation (ρ = 0.23, *p*<0.001) which is perhaps surprising given the often complimentary nature of visual and vestibular processing [37], [38]. Visual biases are generally larger than vestibular ones, particularly for backward movement directions (Fig. 3). Such modality-specific spatial biases may arise at the earliest stages of sensory processing, before signals from different modalities are combined, and have therefore been referred to as encoding biases [34], [35].

For example, overestimation of heading angle that increases with heading eccentricity is predicted by the triangulation strategy of visual heading estimation, whereby the unseen FOE location is estimated by triangulation from two or more optic flow vectors. This is because symmetric error on the direction estimates of individual motion vectors leads to a distribution of FOE estimates that is skewed toward lateral headings [10], [39]–[41]. Overestimation due to this strategy has been observed in a single subject in a previous study in a limited range of +/−10°[41]. Other subjects from the same study exhibited a screen-center bias, similar to previous reports [9], [10]. Such a triangulation strategy could potentially explain overestimation of visual heading in the present study for large heading eccentricities (i.e. >50) where the FOE was located off the screen.

In parallel, modality-specific vestibular biases could arise from differential encoding of fore-aft versus lateral components of linear acceleration stimuli. However, we are not aware of experimental data characterizing such differential properties of the vestibular system. Alternatively, visual and vestibular encoding and estimation may be unbiased. Instead, multimodal spatial processing may have differential effects on visual versus vestibular estimates. At present, these alternative explanations are difficult to evaluate.

One conspicuous difference between visual and vestibular biases is the asymmetry of vestibular biases for backward movement which was not observed in the visual data (Fig. 3). Asymmetric spatial performance has been hypothesized to be related to the right-hemisphere specialization for spatial processing [42] and some have proposed a tight link between this specialization and vestibular function [43]. This may explain why vestibular estimates were more asymmetric than visual ones.

It is also interesting to note that modality-specific biases could potentially explain a puzzling observation in the literature on heading estimation, namely that during combined visual-vestibular heading estimation, vestibular signals are weighted more than predicted based on the standard maximum-likelihood estimation (MLE) model [2], [44], [45]. Most MLE experiments measure cue weights by introducing symmetrical cue conflicts. Under such conditions, the shift of the combined estimate toward one or the other cue provides an empirical measure of cue weights. However, if one cue is biased more than the other, the symmetry is violated, and the shift will depend not only on the weights, but also on the degree of bias. In other words, different degrees of visual and vestibular bias could cause the combined estimate to shift more towards the vestibular cue, and this would be interpreted as vestibular overweighting.

### Conclusion

While some differences were observed across modalities and tasks, the most consistent, novel finding is the overestimation of heading angle relative to straight ahead during forward movements. This overestimation can be predicted based on known properties of neural populations that represent heading information. Such biases could be functionally relevant in terms of providing a high-gain feedback signal for maintaining straight-forward heading during everyday navigation and locomotion.
