# Correction: Systematic Biases in Human Heading Estimation

**DOI:** 10.1371/annotation/38466452-ba14-4357-b640-5582550bb3dd

**Published:** 2013-12-20

**Authors:** Luigi F. Cuturi, Paul R. MacNeilage

The Y-axis labels for Figure 5A and Figure 5B are incorrect. The values should go from -110 (on bottom) to -70 (on top) for Figure 5A and from -95.6 (on bottom) to -55.6 (on top) for Figure 5B. Please see a corrected version of Figure 5 here: http://plosone.org/corrections/pone.0056862.g005.cn.tif

